# Association between DNA methylation in obesity-related genes and body mass index percentile in adolescents

**DOI:** 10.1038/s41598-019-38587-7

**Published:** 2019-02-14

**Authors:** Fan He, Arthur Berg, Yuka Imamura Kawasawa, Edward O. Bixler, Julio Fernandez-Mendoza, Eric A. Whitsel, Duanping Liao

**Affiliations:** 1 Department of Public Health Sciences, the Pennsylvania State University College of Medicine, Hershey, 17033 Pennsylvania USA; 2 Institute for Personalized Medicine, Departments of Biochemistry and Molecular Biology and Pharmacology, the Pennsylvania State University College of Medicine, Hershey, 17033 Pennsylvania USA; 3 Sleep Research and Treatment Center, Department of Psychiatry, the Pennsylvania State University College of Medicine, Hershey, Pennsylvania 17033 USA; 4 Department of Epidemiology, Gillings School of Global Public Health; Department of Medicine, School of Medicine, University of North Carolina at Chapel Hill, Chapel Hill, NC 27599 USA

## Abstract

Childhood obesity remains an epidemic in the U.S. and worldwide. However, little is understood regarding the epigenetic basis of obesity in adolescents. To investigate the cross-sectional association between DNA methylation level in obesity-related genes and body mass index (BMI) percentile, data from 263 adolescents in the population-based Penn State Child Cohort follow-up exam was analysed. Using DNA extracted from peripheral leukocytes, epigenome-wide single nucleotide resolution of DNA methylation in cytosine-phosphate-guanine (CpG) sites and surrounding regions was obtained. We used multivariable-adjusted linear regression models to assess the association between site-specific methylation level and age- and sex-specific BMI percentile. Hypergeometric and permutation tests were used to determine if obesity-related genes were significantly enriched among all intragenic sites that achieved a p < 0.05 throughout the epigenome. Among the 5,669 sites related to BMI percentile with p < 0.05, 28 were identified within obesity-related genes. Obesity-related genes were significantly enriched among 103,466 intragenic sites (P_hypergeometric_ = 0.006; P_permutation_ = 0.006). Moreover, increased methylation on one site within *SIM1* was significantly related to higher BMI percentile (P = 4.2E-05). If externally validated, our data would suggest that DNA methylation in obesity-related genes may relate to obesity risk in adolescents.

## Introduction

Obesity is a well-established risk factor for various chronic diseases, including cerebral vascular disease^1^, cardiovascular diseases (CVD)^2^, and cancer^3^. To date, obesity has been thoroughly studied and considered as a consequence of unhealthy behavioral exposures, such as physical inactivity and excessive energy intake^4,5^. Despite the heightened awareness of these behavioral risk factors, the epidemic of childhood obesity still imposes a major threat to public health in the U.S. According to U.S. Center for Disease Control and Prevention National Center for Health Statistics^6^, the prevalence of childhood obesity was about 17% and affected 12.7 million children and adolescents in 2014. Furthermore, it has been estimated that 40%-70% of the variation in obesity-related phenotypes in humans is heritable^7,8^, suggesting that genetic background also plays a role in obesity. While a large number of obesity-related genes have been identified by genome-wide association studies^9–12^, these genetic studies have only been able to explain a limited proportion of the variance in obesity. For example, a common variant in *FTO*, the strongest obesity-related gene, only explains 1% of the variance in body mass index (BMI)^13^. Therefore, a more thorough understanding of the factors associated with the susceptibility to obesity is needed.

In the last decade, the epigenetic regulation of gene expression has been studied as a novel mechanism that may fill the gap in the etiology of complex disease. In brief, epigenetics is the study of heritable patterns of phenotype resulting from changes in a chromosome without alterations in the DNA sequence^14^. It has been suggested as a molecular mechanism that mediates the interplay between behavioral/lifestyle factors and genetics, by adapting the genome to environmental exposures^15^. Therefore, it is plausible that aberrant epigenetic changes may contribute to the development of human disease. In fact, epigenetic changes, such as DNA methylation, have been associated with the development and progression of chronic disease, including cancer^16^ and CVD^17^.

Although there has been a rapid growth in the field of epigenetics and successes in identifying target sites that relate to obesity, the epigenetic basis of obesity remains largely unknown, particularly in early developmental stages, such as adolescence. Therefore, we carried out this cross-sectional study to explore the epigenetic profile, in particular DNA methylation within obesity related genes, in relationship with marker of general obesity in adolescents. For this report, we assessed peripheral blood leukocyte DNA methylation level, through enhanced, reduced representation bisulfate sequencing (RRBS) assay, and BMI percentile obtained from a population based sample of 263 adolescents who participated the Penn State Child Cohort follow-up exam.

## Results

The major demographic characteristics of the 263 participants with DNA methylation data were summarized in Table 1. The mean (SD) age of the sample was 16.7 (2.2) years. 78.7% of them were non-Hispanic white and 55.9% were male. The prevalence of tobacco use and alcohol drinking were 10.3% and 19.6%, respectively. When stratified according to 4 CDC-defined age- and sex-specific BMI categories^18^, the proportion of underweight, normal weight, overweight, and obese in our sample of adolescents were 1.9%, 61.6%, 20.5%, and 16.0%, respectively. While there was no significant difference in racial distribution across the four age- and sex-specific BMI categories (p = 0.23), the proportion of non-Hispanic white was substantially lower in obese group. Indeed, non-Hispanic white participants showed a significantly lower BMI percentile than participants with other race/ethnicity identity (p = 0.01).

**Table 1:** Demographic and phenotype statistics of the entire study population and stratified by age- and sex-specific BMI categories^a^.

	Overall N = 263	Underweight N = 5	Normal N = 162	Overweight N = 54	Obese N = 42	P value
Age (year)	16.7 (2.2)	17.2 (1.6)	16.6 (2.2)	17.1 (2.6)	16.6 (2.0)	0.45
Male (%)	55.9	60.0	55.6	57.4	54.8	0.99
White (%)	78.7	80.0	80.9	81.5	66.7	0.23
BMI percentile	65.4 (28.5)	2.1 (1.4)	50.7 (22.3)	90.4 (3.0)	97.7 (1.4)	<0.01
SBP (mm Hg)	114.0 (11.9)	105.7 (5.0)	112.0 (11.3)	116.9 (13.2)	118.7 (10.8)	<0.01
DBP (mm Hg)	66.7 (9.1)	65.0 (9.9)	65.2 (8.8)	69.4 (9.8)	69.2 (8.1)	<0.01
Family History						
Obesity	30.8	0.0	21.0	38.9	61.9	<0.01
Hypertension	36.5	60.0	33.3	31.5	52.4	0.12
Diabetes	11.4	0.0	9.9	11.1	19.1	0.09

Data were presented as mean (SD) and proportions for continuous and categorical variables, respectively.

^a^Defined by U.S. CDC. Underweight: BMI percentile <5; Normal weight: 5 ≤ BMI percentile <85; 85 ≤ Overweight <95: Obese: BMI percentile ≥ 95.

**Figure 1:**
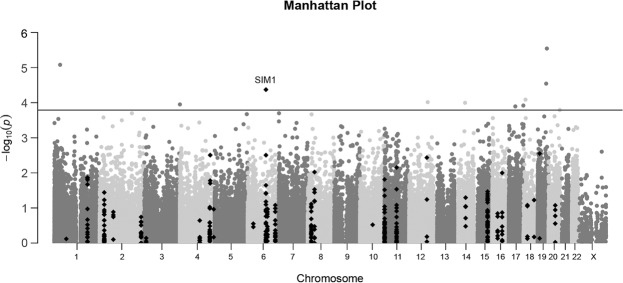
Manhattan plot for genome-wide p values for the association. P values were obtained by linear regression analysis with adjustment for age, race, and sex, and batch effect. The y axis shows the −log_10_(p) of 103,466 sites, and x axis shows their chromosome position. The red dots represent the sites on obesity-related genes. The horizontal red line represents the threshold for significance within obesity-related sites (p = 1.6E-04). None of the sites was significant at genome-wide level (p = 4.8E-07).

Among the 166,158 analyzable cytosine-phosphate-guanine (CpG) sites, 165,297 (99.5% of) the sites were successfully analysed in linear regression models. After excluding intergenic sites, 103,466 intragenic sites were annotated onto 11,490 unique genes. The Manhattan plot showing the genome-wide p values of the association between DNA methylation level on individual CpG site and BMI percentile is provided in Fig. 1. The 20 most significant site were presented in Supplementary **(**see Supplementary Table S1**)**. As noted, none of the site passed the threshold for genome-wide significance (p < 4.8E-07), with an estimated genomic inflation factor of 1.04. Further looking into all intragenic sites, 5,669 were associated with BMI percentile at p < 0.05 level. Among them, 28 were on 11 obesity-related genes. In fact, one of the site was the 4^th^ most significant hit (p = 4.2E-05) throughout the entire epigenome. The distribution of sites based on their relationship with obesity genes were summarized in Table 2. Briefly, among the 103,466 intragenic sites, 308 were on obesity-related genes, 9.1% (N = 28) of them were significant at p < 0.05 level, while in sites not related to obesity genes (N = 103,158), 5.5% (N = 5,641) were achieved a p < 0.05. Both hypergeometric and permutation test P values (0.006 and 0.006, respectively) suggested that the obesity-related genes were significantly enriched.

**Table 2:** Distribution of significant sites according to obesity and non-obesity related genes.

		On an Obesity Gene		Total (N)
		Yes (N)	No (N)	
P < 0.05	Yes (N)	28	5,641	5,669
	No (N)	280	97,517	97,797
Total (N)		308	103,158	103,466

We further examined the relationship between site-specific methylation level within obesity-related genes, which was obtained through a query of gene-disease association database (DisGeNET)^19^, and BMI percentile. The top 15 most significant sites within obesity-related genes were summarized in Table 3. As shown in the Table 2 sites were within *SIM1* gene. More importantly, there was 1 site (Chr6: 100903612), which is on CpG:24 of *SIM1*gene, that remained significantly related to BMI percentile, even after applying a Bonferroni (p = 4.2E-05) or false discovery rate adjustment (q = 0.013). The regression coefficient (SE) between a 10% increase in methylation in this site and BMI percentile was 7.2 (1.7).

**Table 3:** Top 15 significant sites^a^ in association between 10% increase in DNA methylation level and BMI percentile.

Chromosome	Position	Gene Symbol	β	(SE)	P value	Function/Disease^b^
chr6	100903612	SIM1	7.2	1.7	4.2E-05	Energy Homeostasis
chr19	10386701	ICAM1	−38.7	12.8	2.8E-03	Early Inflammation
chr4	166414631	CPE	4.0	1.3	3.1E-03	Biosynthesis of Insulin
chr6	100903599	SIM1	3.8	1.3	3.1E-03	Energy Homeostasis
chr12	97906654	RMST	−4.4	1.5	3.7E-03	Early Onset Obesity
chr11	65837921	PACS1	191	70	7.0E-03	Severe Obesity
chr8	37822759	ADRB3	3.7	1.4	9.5E-03	Lipolysis/Thermogenesis
chr16	53738226	FTO	37.3	14.0	1.0E-02	Obesity
chr1	182360830	GLUL	7.9	3.2	1.4E-02	Insulin Secretion
chr1	182360849	GLUL	8.0	3.2	1.4E-02	Insulin Secretion
chr1	182360819	GLUL	7.9	3.2	1.4E-02	Insulin Secretion
chr10	135342166	CYP2E1	4.5	1.8	1.5E-02	Gluconeogenesis
chr1	182360834	GLUL	7.8	3.2	1.6E-02	Insulin Secretion
chr4	166414620	CPE	29.3	12.1	1.7E-02	Biosynthesis of Insulin
chr4	166414611	CPE	29.0	12.2	2.0E-02	Biosynthesis of Insulin

^a^All sites were mapped to hg19 assembly.

^b^Gene Function/Disease were supplied by RefSeq or OMIM and summarized in NCBI.

## Discussion

Although there is an increasing awareness of lifestyle and environmental factors associated with obesity, major gaps still exist in understanding the contribution of epigenetics to the risk of obesity, especially in otherwise healthy adolescents. As obesity is a complex and multifactorial-determined disease, it is highly plausible that BMI is regulated by epigenetic mechanisms in multiple related genes. Since the 1980’s, a large body of evidence supporting the role of DNA methylation as a major epigenetic regulator of gene expression has been obtained^20–27^. Therefore, the primary aim of this study was to assess the overall association between DNA methylation and BMI percentile. Our enrichment analyses results, derived from a representative cohort of central Pennsylvania adolescents, suggest that an aggregated change of methylation levels in obesity-related genes is significantly associated with obesity in adolescents free of cardiometabolic disease manifestations. This finding confirmed, and more importantly, extended the understanding of the association between DNA methylation and obesity, since a majority of previous studies reported the association based on studies in newborns or adult populations^19–23^, participants with clinically diagnosed morbidities^23,24^, or interventional studies^25–27^. For example, Dick *et al*. reported that DNA methylation in whole-blood DNA was related to BMI in a group of middle-aged myocardial infarction survivors and healthy blood donors^22^. In another sample of 25 obese men, Milagro and coworkers reported that weight loss was associated with DNA methylation in peripheral blood mononuclear cells in an 8-week nutritional intervention^25^. We, for the first time, observed that DNA methylation levels in obesity genes are related to BMI percentile in otherwise healthy adolescents. Such an association may operate mainly through energy homeostasis (e.g. *SMI1* and *CPE*) and lipid mobilization (e.g. *ADRB3*). While methylation level in the individual genes may be weakly related to BMI percentile in adolescents, in aggregate, the enrichment analysis demonstrated a significant relationship. The collective change in methylation profiles in obesity-related genes may well lead to obesity.

Recently, a large amount of epigenetic markers have been reported in both adult^28,29^ and children^30–32^ in epigenome-wide association studies (EWAS). Wahl *et al*. in a recent meta-analyses demonstrated that BMI was associated with DNA methylation changes in 187 genetic loci in adults^28^. Later, Sayols-Baixeras *et al*. discovered that methylation level in 70 CpGs and 33 CpGs related to BMI and waist circumferences in middle-aged adults and elderly^29^. In a case-control study between severely obese children and lean controls, Fradin and coworkers found 10 CpGs sites with a difference in methylation greater than 10%^30^. In another two studies in children, Ding *et al*. found a total of 852 differentially methylated CpGs in Chinese population^31^, while Huang *et al*. identified 129 differentially methylated CpGs among children seeking treating for obesity in Western Australia^32^. However, none of the CpGs was consistently associated with obesity across these studies, which indicated substantial false positive findings may exist in these EWAS studies. Therefore, our secondary goal of the current study was to identify individual sites, whose methylation level is related to obesity, with focus on sites within genes that known to be associated with obesity. Unlike the previous EWAS studies, none of the sites was significantly related to obesity at genome-wide level. The difference in our results and previous studies may due to the differences in the genetic background of the population and study design. However, we found one novel site in *SIM1* that was significantly associated with BMI percentile when focusing on obesity-related genes even after Bonferroni correction. To the best of our knowledge, this is the first time that *SIM1*methylation has been associated with obesity in adolescents.

*SIM1* plays a key role in neuronal differentiation within the paraventricular nucleus of hypothalamus, which is critical for food intake regulation^33^. Data from both mice^34^ and human^35,36^ studies have shown that insufficiency of *SIM1* is related to obesity and Prader-Willi-Like (PWL) syndrome. Bonnefond *et al*. demonstrated a firm link between *SIM1* loss-of-function mutations, severe obesity, and PWL features in 1,193 children^36^. Therefore, hyper-methylation in *SIM1*, which may result in a repression of gene expression and consequently *SIM1* insufficiency, could relate to obesity. Indeed, our analyses showed that increased methylation level on 1 site (Chr6: 100903612) in *SIM1* was significantly associated with BMI percentile. Specifically, with 10% increase in methylation level, the corresponding increase in BMI percentile was 7.2 (1.7). While validation is pending, our result provided a potential target site for future studies investigating DNA methylation and obesity. Meanwhile, it should be noted that discovery studies, including the current one, may overestimate the strength of the association. Also, despite previous evidence suggested that DNA methylation in *SIM1* may be predictive of obesity, it is also biologically plausible that alternation in DNA methylation was driven by presence of obesity. Therefore, more longitudinal studies are warranted.

The modifiable factors responsible for the variation in methylation levels among obesity-related genes are pending further investigations. One of the hypothesized origins of this variation includes dietary/lifestyle factors in the life course^37^. For example, in a meta-analysis of 13 cohorts (N = 6,685) of newborns, Joubert and coworkers reported that a large number of sites responded to maternal smoking in pregnancy^38^. Tsaprouni *et al*. observed that cigarette smoking altered DNA methylation level at multiple sites in middle-aged adults, and the effect might be partially reversible upon cession^39^. Moreover, previous data has shown that dietary and physical activity interventions^25–27,40^ could induce changes in DNA methylation levels in genes related to obesity. On the other hand, environmental exposures have also been postulated as a contributor to the variation in DNA methylation^41^. Specifically, ambient air pollution and heavy metal exposure may trigger changes in DNA methylation at both genome-wide^42–44^ and gene levels^45–47^, with the time-course of the effects ranging from hours to months. For example, two articles based on Normative Aging Study showed that both rapid^45^ and prolonged^46^ air pollution exposure are associated with global DNA methylation level changes. Despite cumulating efforts to investigate the factors affecting DNA methylation level, more studies of variation in methylation levels in obesity-related genes among healthy adolescents are needed.

Compared to previous studies exploring the relationship between DNA methylation and obesity, several strengths of our current study may be noted. Our study is the first to investigate such a relationship in a representative sample of adolescents from general population. Majority of the previous studies were conducted in populations in other age groups^20–24^ or individuals with cardiovascular and metabolic morbidities^25,26^. As age and cardiovascular health may substantially change the DNA methylation landscape, those results may not be extrapolated to healthy adolescents. Our analyses on DNA methylation level also were based on linear regression models, not case-control comparative approaches. By utilizing the linear regression model, we preserved the statistical power of our data and avoided the dilemma of arbitrarily establishing a threshold for “differential methylation”. In addition, analyses performed in adolescents from the extremes of the obesity distribution^30–32,48^ are unlikely to reflect the association between DNA methylation and obesity in the general population as a whole. Therefore, results from our study may have wider public health implications. Furthermore, our high throughput assay generated genome-wide site-specific methylation levels that were leveraged in the identification of a novel site in *SIM1* that was significantly related to BMI percentile. This result may contribute to the current collection of “targeted sites” for future studies.

While the current study possesses the strengths stated above, its limitations shall not be overlooked. First, there were extensive missing data in the raw methylation sequencing data, which is a result of using a novel whole-genome methylation sequencing technique on the low-yield DNA samples. To maintain the validity of our results, we purposely excluded those sites with <10x coverage or available from <50% of the study population. In the enrichment analyses, we further limited our scope on CpG sites intragenic to obesity genes. However, we did not find a statistically significant bias associated with the missing data when comparing BMI percentile in the missing and non-missing groups using a 5% level two-sample Mann-Whitney U test on the 15 sites presented in Table 3. Nevertheless, the degree of missing methylation data has substantially reduced the number of analyzable sites. Second, the number of DNA samples was relatively small. With 263 DNA samples, our statistical power to detect significant a relationship between DNA methylation and BMI percentile is limited, especially at genome-wide level. More importantly, our ability to control for assay-related covariables, particularly cell type mixture, was severely restricted. Our small sample size also prevented us from dividing the entire cohort into discover and validation cohorts. Therefore, external validation is needed to confirm our results. However, both gene-set based and site-based analyses, independently, showed a significant relationship between DNA methylation and BMI percentile. The internal consistency of these results boosts confidence in the findings. Third, there is little previous functional work and evidence confirms that methylation in *SIM1* would result in an absence of *SIM1* signaling. However, one can postulate that hyper-methylation of *SIM1* would suppress its expression. As it has been proved that haploinsufficiency of *SIM1* is related to obesity in genetic studies^34–36^, it is plausible that methylation in *SIM1* may contribute to reduced *SIM1* expression, and through which related to obesity. Fourth, alcohol drinking and cigarette smoking were not controlled for in the analyses. However, since the vast majority of our study population was under legal alcohol drinking age and/or minimum age of purchasing cigarettes, drinking and smoking behaviors obtained through a self-administered questionnaire, are highly likely to be biased. Lastly, the current analyses were based on cross-sectional data. Hence, causal inference cannot be made. In fact, in a recently published paper^28^, Wahl *et al*. used genetic association analyses to demonstrate that the alterations in DNA methylation are predominately the consequence of obesity, rather than the cause. However, one could argue that failures in gene imprinting, due to aberrant DNA methylation changes, in particular on those genes affecting growth factors or regulators of genes controlling growth and energy metabolism, is the underlying mechanism linking DNA methylation and obesity^8^.

In conclusion, we observed that aggregated change in methylation of DNA from peripheral blood leukocytes on obesity-related genes was associated with obesity in a population-based sample of healthy adolescents. Although validation is pending, we further identified a novel site on *SIM1*, whose DNA methylation level is related to obesity. Taken together, this evidence suggests that DNA methylation change is related to obesity, even in healthy adolescents. Such an early life change may be associated with elevated cardiometabolic risk in adulthood. However, further longitudinal studies with larger sample sizes are warranted. Such studies may be used to confirm our findings and provide deeper insight into the causal direction of the association, as well as possible origins of the variation in DNA methylation levels.

## Materials and Methods

### Population

For this analysis, we used data from participants in the Penn State Child Cohort follow-up examination. The recruitment and examination procedures for the baseline^49^ and follow-up^50^ examinations have been published elsewhere. Briefly, a total of 700 children aged 5–12 years were recruited from central Pennsylvania and participated in the baseline examination in 2002–2006. 421 of them participated in the follow-up examination in 2010–2013, with an average of 7.70 years of follow-up. In the follow-up examination, each participant underwent a standardized physical examination, in which the participant’s height and weight were measured by a trained investigator. BMI was calculated as weight/height^2^ (kg/m^2^). Then BMI was converted to BMI percentile in accordance with U.S. Centers for Disease Control and Prevention’s guideline and algorithm^51^. Participants’ major demographic characteristics, including age, race, sex, tobacco use, and alcohol drinking were collected via a self-administered questionnaire. Parents’ history of obesity, diabetes, and hypertension were also obtained.

The study protocol was approved by Penn State University College of Medicine Institutional Review Board. All research was performed in accordance to relevant guidelines/regulations. Written informed consents were obtained from participants and their parents or legal guardians if younger than 18 years.

### Genome-wide Methylation Assay

391 of the 421 participants in the follow-up study consented for blood draw. Fasting peripheral blood samples were collected from each participant and stored at −80 °C until use. Among the 391 blood samples, 263 yielded adequate amount of DNA were sequenced. There was no significant difference in the major demographic characteristics between the subjects whose DNA were sequenced and the original cohort **(**see Supplementary Table S2**)**. DNA from peripheral blood leukocyte was extracted and subjected to RRBS using a modified method. Single nucleotide resolution of DNA methylation in CpG sites and surrounding regions were detected using Illumina HiSeq. 2500. This highly sensitive, multiplexed method generated a specific, reduced representation of the genome of DNA fragments enriched for CpG dinucleotides. Briefly, genomic DNA (minimum 5 ng) was digested with a methylation-insensitive restriction enzyme, MspI, which recognizes CCGG. The digested DNA fragments were purified and subjected to adapter ligation and size selection by AMPure magnetic beads (Beckman Coulter Inc., Brea, CA, USA.). The resulting libraries, covering the target size range between 40 to 200 bp, were quantified by Kapa Library Quantification Kit (Kapa Biosystems Inc., Wilmington, MA, USA). Equimolar libraries were pooled and unmethylated cytosines (C) were converted to uracils (U) with bisulfite, amplifed by polymerase chain reaction, and then sequenced. The degree of methylation of each fragment, estimated from the number of converted reads compared to the unconverted reas in each CpG, was calculated. Base calls of bisulfite treated sequencing reads with phred quality scores <20 and/or length <35 bp were trimmed and the adaptor was cut using trim_galore V0.3.3 (Babraham Bioinformatics, Cambridge, UK). Resulting reads were mapped to the hg19 assembly and methylation calls were performed using Bismark v0.10.1 (Babraham Bioinformatics, Cambridge, UK). Approximately 1.6 million methylation levels were detected at more than 10x coverage for each subject.

### Statistical analyses

The demographic and phenotypic characteristics of the entire cohort, as well as stratified by four CDC defined age- and sex-specific BMI categories^18^, were summarized as mean (SD) and proportions for continuous and categorical variables, respectively. ANOVA and Cochran-Mantel-Haenszel test were used to compare the distribution of these characteristics, as appropriate.

To ensure the validity of the methylation data and statistical inferences of the analyses, we excluded bases with <10x coverage or available from <50% (N = 132) of the subjects, leaving a total of 166,158 analyzable sites. We used multi-variable adjusted linear regression models to investigate the relationship between DNA methylation and BMI percentile. In the models, BMI percentile and site-specific methylation levels were treated as dependent and independent variables, respectively, while adjusting for age, race, sex, and batch of assay. Sites from the converged models were annotated based on the hg19 assembly. All intragenic sites were then taken forward for enrichment analyses.

From a query of DisGeNET^19^, a database of gene-disease associations, we identified a list of fifty obesity related genes (Table 4) using a reliability score threshold (≥0.20). The intragenic sites were subsequently categorized into obesity and non-obesity-related sites. Gene-set enrichment was assessed with a hypergeometric test and a permutation test^52^ with 10,000 permutations. These tests compared the distribution of CpG sites, whose methylation level associated with BMI percentile at p < 0.05 level intragenic to obesity-related and non-obesity related genes. A p value < 0.05 was used to determine the significance of the enrichment analysis.

**Table 4:** List of top 50 obesity-related genes^a^.

Gene Name	Score of Reliability	Gene Name	Score of Reliability
*MC4R*	0.79	*ESR1*	0.23
*LEPR*	0.64	*MC3R*	0.23
*LEP*	0.44	*UCP2*	0.22
*PPARG*	0.44	*IRS1*	0.22
*FTO*	0.40	*HSD11B1*	0.22
*ADRB3*	0.34	*SIRT1*	0.21
*ADRB2*	0.33	*F2*	0.21
*UCP3*	0.31	*SIM1*	0.21
*ADIPOQ*	0.30	*AGRP*	0.21
*IL6*	0.29	*NPY5R*	0.21
*NPY1R*	0.28	*CYP2E1*	0.20
*ENPP1*	0.28	*ICAM1*	0.20
*SH2B1*	0.28	*CPE*	0.20
*CARTPT*	0.26	*SERPINA12*	0.20
*NPC1*	0.25	*PMCH*	0.20
*LPL*	0.25	*LBP*	0.20
*POMC*	0.25	*ACSL1*	0.20
*HOXB5*	0.24	*GPX3*	0.20
*PRKCH*	0.24	*HK2*	0.20
*PACS1*	0.24	*SIRT3*	0.20
*RMST*	0.24	*FOXO3*	0.20
*TNF*	0.24	*GLUL*	0.20
*CNR1*	0.23	*HADH*	0.20
*INS*	0.23	*TF*	0.20
*PCSK1*	0.23	*NEIL1*	0.20

^a^Data retrieved from DisGeNET v4.0 (http://www.disgenet.org/web/DisGeNET/menu/browser/tab8b?3&pview=default&pf=http://www.disgenet.org/web/DisGeNET%3Fdata/diseases::umls:C0028754::de&pf=/data/sources::ALL::de), Integrative Biomedical Informatics Group GRIB/IMIM/UPF. [May. 2017].

**Figure 2:**
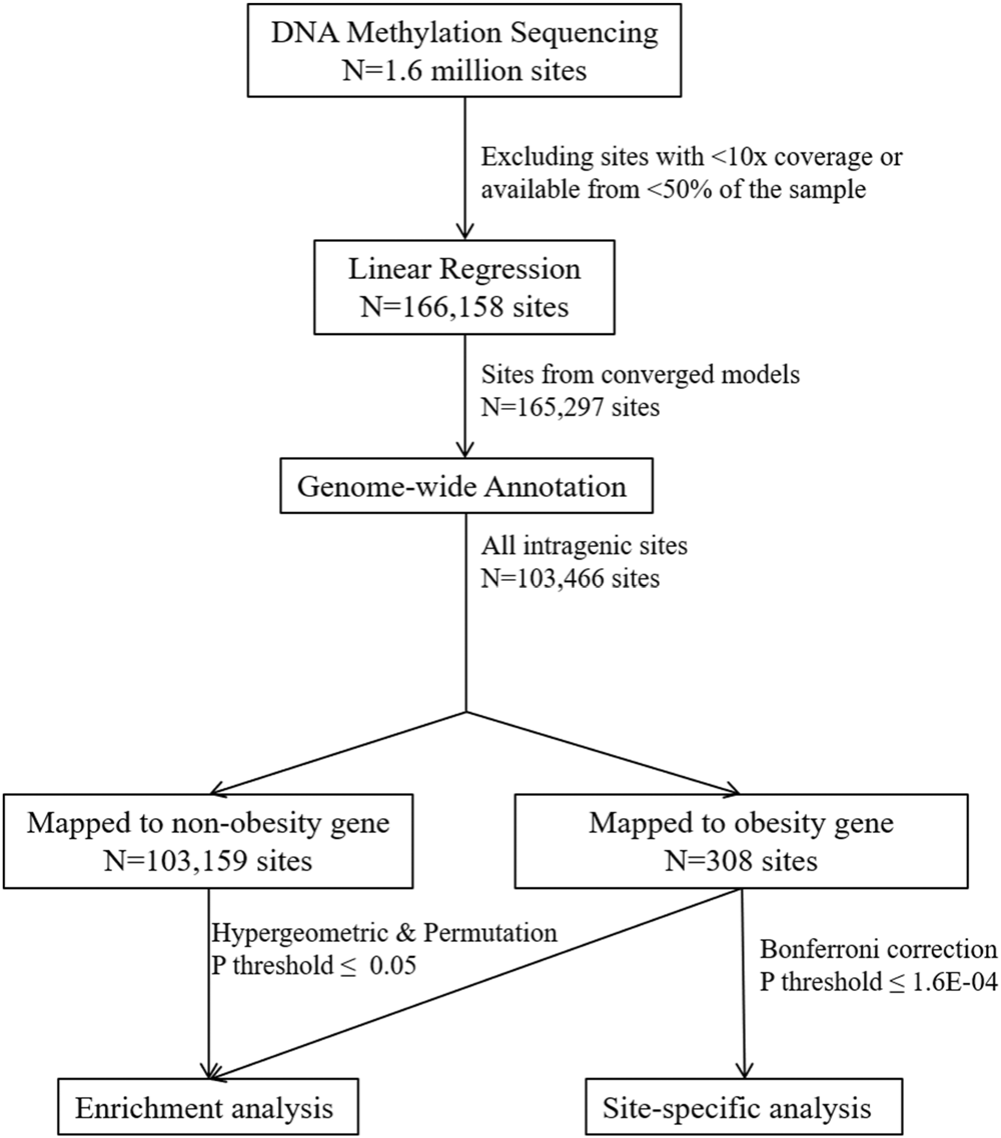
Analytic diagram.

To identify individual sites, particularly those intragenic to obesity-related genes that were significantly related to BMI percentile, Bonferroni correction was used to account for multiple testing. A genomic inflation factor was also calculated to assess the degree of bulk inflation and the excess false positive rate^53^. All analyses were performed by using R (version 3.3.0). The entire analytic process was summarized in a diagram (Fig. 2) to graphically illustrate our statistical approaches.

## Supplementary information

Supplementary Tables

## Data Availability

The datasets generated during and/or analysed during the current study are available from the corresponding author on reasonable request.
