# Supplementary material for: Association between DNA methylation in obesity-related genes and body mass index percentile in adolescents
Source: Sci Rep. 2019 Feb 14;9:2079. doi: 10.1038/s41598-019-38587-7 (PMC6375997; doi:10.1038/s41598-019-38587-7)
Supplement: Supplementary file 1 — Supplementary Tables [file 41598_2019_38587_MOESM1_ESM.pdf]

Association between DNA methylation in obesity-related genes and body mass index percentile in adolescents

Fan He[1], Arthur Berg[1], Yuka Imamura Kawasawa[2], Edward O. Bixler[3], Julio Fernandez-Mendoza[3], Eric A. Whitsel[4], Duanping Liao[1*]

1. Department of Public Health Sciences, the Pennsylvania State University College of Medicine, Hershey, 17033, Pennsylvania, USA.

2. Institute for Personalized Medicine, Departments of Biochemistry and Molecular Biology and Pharmacology, the Pennsylvania State University College of Medicine, Hershey, 17033, Pennsylvania, USA.

3. Sleep Research and Treatment Center, Department of Psychiatry, the Pennsylvania State University College of Medicine, Hershey, Pennsylvania, 17033, USA

4. Department of Epidemiology, Gillings School of Global Public Health; Department of Medicine, School of Medicine, University of North Carolina at Chapel Hill, Chapel Hill, NC, 27599, USA.

Supplementary Table S1. Top 20 genome-wide significant sites in association between 10% increase in DNA methylation and BMI percentile.

Chromosome	Position	Gene Symbol	P value	Function/Disease[1]
chr19	50096127	PRR12	2.9E-06	Nervous system development
chr1	32083651	HCRTR1	8.3E-06	Regulation of feeding behavior
chr19	46307719	RSPH6A	2.9E-05	Primary ciliary dyskinesia-2
chr6	100903612	SIM1	4.3E-05	Energy Homeostasis
chr18	10777054	PIEZO2	8.2E-05	Type 5 distal arthrogryposis
chr12	102271631	DRAM1	9.7E-05	Tumor suppressor pathway
chr14	58618976	ARMH4	1.0E-04	N/A
chr3	197714006	LMLN	1.1E-04	Cell migration and invasion
chr17	79979278	CENPX	1.2E-04	Resistance of DNA damage
chr17	35850659	DUSP14	1.3E-04	Signaling pathways
chr20	61886741	FLJ16779	1.6E-04	N/A
chr7	1571105	MAFK	2.0E-04	Globin gene expression regulation
chr2	177054528	HOXD1	2.0E-04	Limb development
chr5	176278673	UNC5A	2.1E-04	Apoptosis
chr8	21584425	GFRA2	2.2E-04	Neuron survival and differentiation
chr20	33264503	PIGU	2.4E-04	Cell division control
chr19	35633487	FXYD1	2.5E-04	Ion channel activity
chr2	21022251	LDAH	2.6E-04	Cholesterol mobilization
chr16	2581986	MIR3178	2.8E-04	Translational inhibition
chr18	11982237	IMPA2	2.8E-04	Bipolar disorder

Gene Function/Disease were supplied by RefSeq or OMIM and summarized in NCBI.

P threshold for genome-wide significance: < 4.8E-07

Supplementary Table S2. Comparison of demographic characteristic between baseline cohort and subjects with DNA sequencing.

	**Baseline N=700**	**DNA Sequenced N=263**	**P value**
Age (years)	9.2 (1.7)	16.7 (2.2)	<0.001
Male (%)	52.9	55.9	0.40
White (%)	76.3	78.7	0.43
BMI percentile	63.0 (29.0)	65.4 (28.5)	0.77
BMI category[1] (%)			
Underweight	2.4	1.9	0.55
Normal	65.6	61.6	
Overweight	14.4	20.5	
Obese	17.6	16.0	

Data are presented as mean (SD) and proportions for continuous and categorical variables, respectively. T-test, chi-square test, and Cochran–Mantel–Haenszel test were used to compare the distributions between baseline cohort and subjects with DNA sequenced, as appropriate.

1. Age- and sex-specific BMI categories were defined by U.S. CDC. Underweight: BMI percentile < 5; Normal weight: 5 ≤ BMI percentile < 85; 85 ≤ Overweight < 95: Obese: BMI percentile ≥ 95.
